# Rigler’s Triad: A Radiological Sign of Gallstone Ileus

**DOI:** 10.31662/jmaj.2024-0192

**Published:** 2024-11-01

**Authors:** Ryohei Ono, Izumi Kitagawa

**Affiliations:** 1 Department of Cardiovascular Medicine, Chiba University Graduate School of Medicine, Chiba, Japan; 2 Department of General Internal Medicine, Shonan Fujisawa Tokushukai Hospital, Fujisawa, Japan

**Keywords:** cholecystoduodenal fistula, chronic cholecystitis, gallstone ileus, pneumobilia, Rigler’s triad

A 100-year-old woman with a history of cholelithiasis and chronic cholecystitis presented with a 5-hour history of left flank pain and repetitive vomiting. Physical examination revealed abdominal tenderness in the left flank region without guarding. Abdominal computed tomography (CT) scans showed pneumobilia, a gallstone (20 mm × 30 mm) in the small intestine, and small-bowel obstruction (Rigler’s triad) (Figure 1). The abdominal CT scans 1 year prior showed a gallstone in the gallbladder adjacent to the duodenum (Figure 2); therefore, the gallstone passed through the cholecystoduodenal fistula owing to repeated chronic cholecystitis. She was diagnosed with gallstone ileus. Laparotomy was performed, and the gallstone was finally removed (Figure 3). Her postoperative course was uneventful.

**Figure 1.**
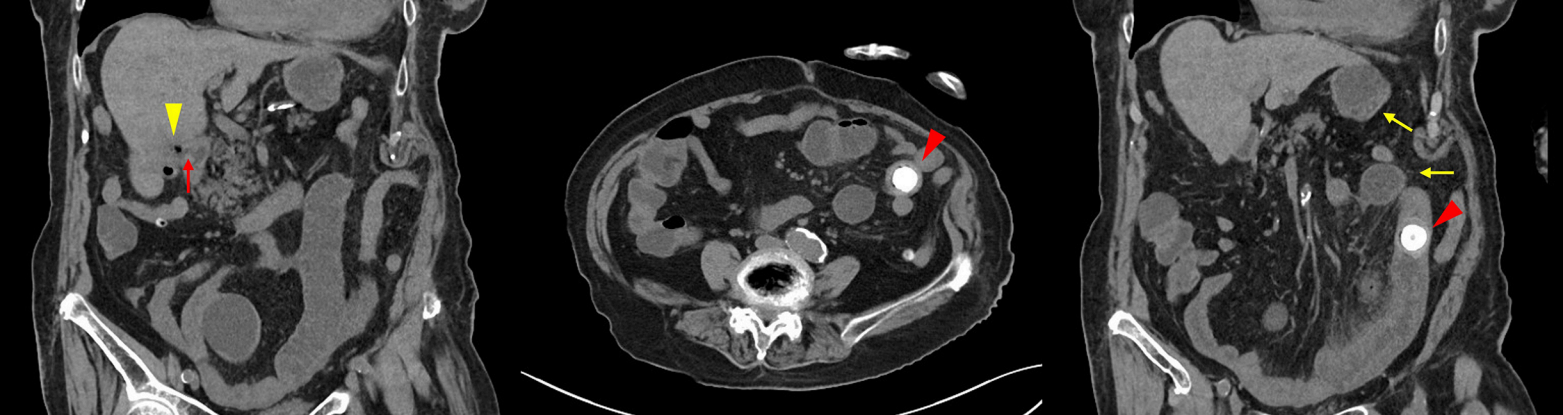
Abdominal computed tomography scans on the present visit showing pneumobilia (yellow arrowhead), the cholecystoduodenal fistula (red arrow), a gallstone (red arrowheads) in the small intestine, and small-bowel obstruction (yellow arrows).

**Figure 2.**
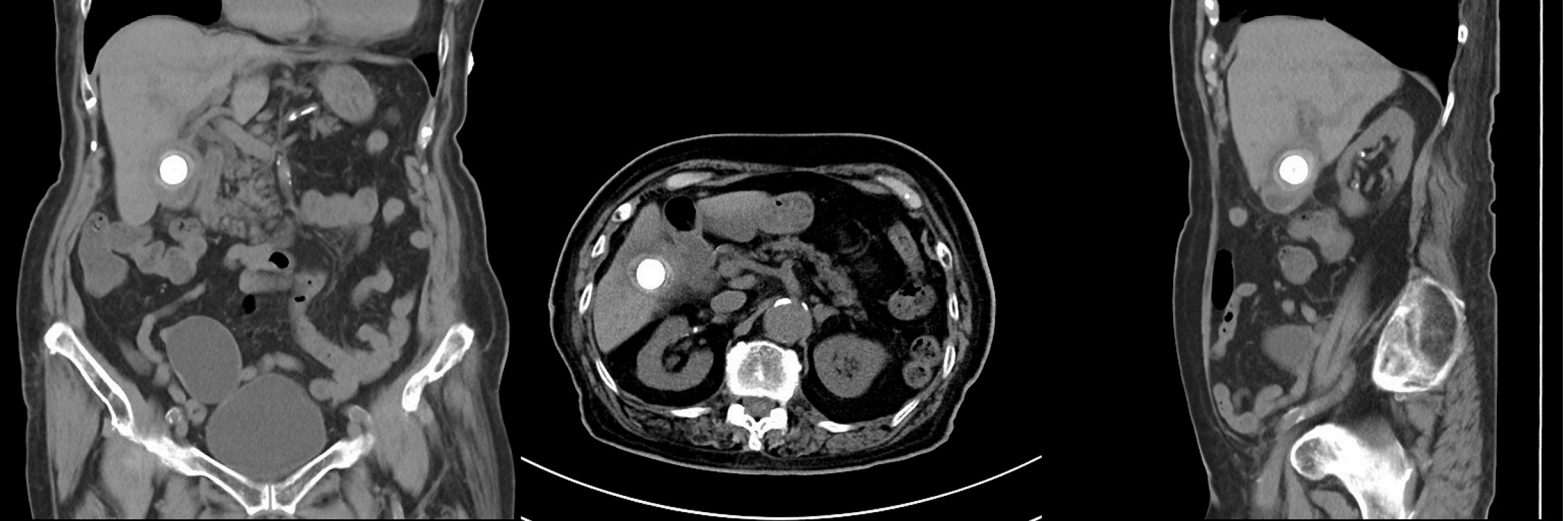
Abdominal computed tomography scans 1 year prior showing the gallstone in the gallbladder adjacent to the duodenum.

**Figure 3.**
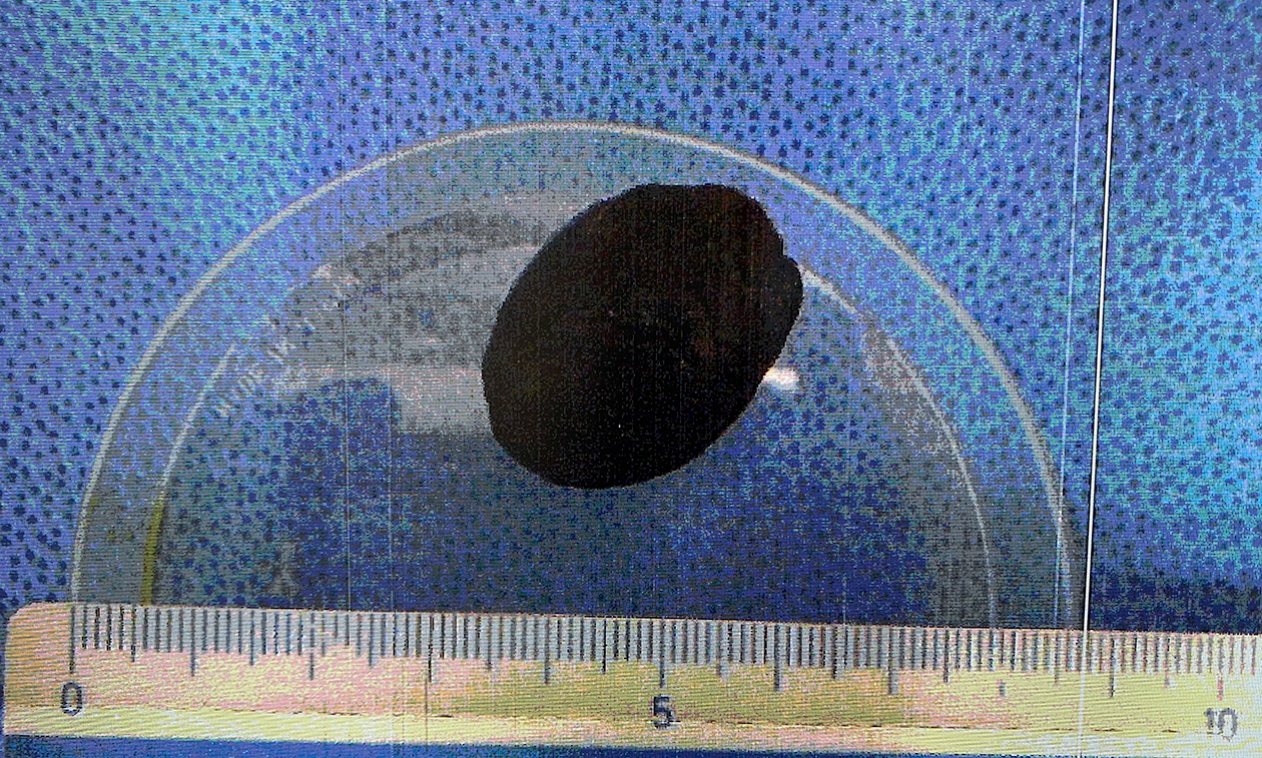
Gallstone removal by laparotomy.

Gallstone ileus is a mechanical obstruction caused by a gallstone passing through a cholecystoduodenal or cholecystogastric fistula ^(1)^. Rigler’s triad is a highly specific radiological finding of gallstone ileus that includes pneumobilia, small-bowel obstruction, and ectopic gallstone ^(2)^.

## Article Information

### Conflicts of Interest

None

### Author Contributions

Ryohei Ono: Writing - Original draft, Methodology

Izumi Kitagawa: Methodology, Writing - review and editing

All authors have read and approved the final version of the manuscript.

### Approval by Institutional Review Board (IRB)

IRB approval was not required for this study.

### Informed Consent

Consent was obtained from the patient for using images for publication.
